# Antioxidants of Amaranth, Quinoa and Buckwheat Wholemeals and Heat-Damage Development in Pseudocereal-Enriched Einkorn Water Biscuits

**DOI:** 10.3390/molecules27217541

**Published:** 2022-11-03

**Authors:** Lorenzo Estivi, Luisa Pellegrino, Johannes A. Hogenboom, Andrea Brandolini, Alyssa Hidalgo

**Affiliations:** 1 Department of Food, Environmental and Nutritional Sciences (DeFENS), Università degli Studi di Milano, Via Celoria 2, 20133 Milan, Italy; 2 Consiglio per la Ricerca in Agricoltura e L’analisi Dell’economia Agraria–Centro di Ricerca Zootecnia e Acquacoltura (CREA-ZA), Viale Piacenza 29, 26900 Lodi, Italy

**Keywords:** amino acids, carotenoids, colour, furosine, polyphenols, protein, tocols, pseudocereals

## Abstract

A viable approach to improve the nutritional quality of cereal-based foods is their enrichment with pseudocereals. The aim of this research was to evaluate the antioxidant properties of amaranth, quinoa and buckwheat, and the heat damage of water biscuits (WB) produced from either wholemeal or refined flour of einkorn and enriched with 50% buckwheat, amaranth or quinoa wholemeal. Buckwheat had the highest tocols content (86.2 mg/kg), and einkorn the most carotenoids (5.6 mg/kg). Conjugated phenolics concentration was highest in buckwheat (230.2 mg/kg) and quinoa (218.6 mg/kg), while bound phenolics content was greatest in einkorn (712.5 mg/kg) and bread wheat (675.7 mg/kg). The all-wholemeal WB had greater heat damage than those containing refined flour (furosine: 251.5 vs. 235.8 mg/100 g protein; glucosylisomaltol: 1.0 vs. 0.6 mg/kg DM; hydroxymethylfurfural: 4.3 vs. 2.8 mg/kg DM; furfural: 8.6 vs. 4.8 mg/kg DM). The 100% bread wheat and einkorn wholemeal WB showed greater heat damage than the WB with pseudocereals (furfural, 9.2 vs. 5.1 mg/kg; glucosylisomaltol 1.1 vs. 0.7 mg/kg). Despite a superior lysine loss, the amino-acid profile of the pseudocereals-enriched WB remained more balanced compared to that of the wheats WB.

## 1. Introduction

Bread wheat and durum wheat are the main ingredients of an ever-increasing number of cereal-based foods. However, there are other crops which, besides being valid alternatives to wheat cropping in marginal soils, may contribute to the improvement of the nutritional quality of the finished products. Three pseudocereals, i.e., amaranth (*Amaranthus* spp. L.), quinoa (*Chenopodium quinoa* Willd.) and buckwheat (*Fagopyrum esculentum* Moench), stand out for the well-balanced amino-acid profile of their proteins [1] and the presence of minerals, fibres and antioxidants [2,3,4,5]. Buckwheat, a crop originating from the Eastern side of the Himalayas and south-west China [6], is widely distributed in the temperate areas of the world. Amaranth and quinoa, from Central America and the Andean region of South America, until recently had a more restricted area, but increased demand and insufficient supply pushed their growth to many new areas [7].

As mentioned above, pseudocereals are a good source of antioxidant compounds, such as tocols, carotenoids, phenolic acids, flavonoids, etc., which garner considerable interest for their effect on the reduction in the incidence of aging-related and chronic ailments such as cancer and neurodegenerative or cardiovascular diseases [8]. Pseudocereals can be employed to enrich traditional foods or to manufacture new products. However, they lack the dough-forming gluten needed for the preparation of many popular foods (e.g., bread, bakery products, pasta, etc.) with good textural and sensorial properties. On the other hand, they can be added to wheat flour to improve the nutritional properties of final products, although excessive quantities may lead to technological challenges and mediocre quality of foods, mainly due to the diluting of the gluten. Widely consumed products such as bakery products and pasta are optimal vehicles for the inclusion of nutrients and are, thus, increasingly employed for the prevention and the management of health disorders [9].

Among bakery products, cookies, biscuits and crackers—characterised by a broad array of shapes, enticing taste and long shelf-life—are extremely popular. Crackers are thin and crisp products manufactured from unsweetened and unleavened dough. As such, they are a perfect model product to evaluate the effects of the addition of nutritionally valuable compounds. Their preparation, which requires baking at 200 °C or higher temperatures, induces many chemical reactions, including caramelisation and the Maillard reaction. Caramelisation, favoured by low moisture and temperatures above 120 °C, is a consequence of carbohydrates degradation [10]. The Maillard reaction, instead, takes place at intermediate moisture and temperatures above 50 °C, between reducing sugars and free amino groups: the formation of chemically stable and nutritionally unavailable lysine derivatives leads to a loss of this essential amino acid.

Specific heat-damage indices, such as furosine, hydroxymethylfurfural (HMF), glucosylisomaltol (GLI) and furfural, as well as colour changes, may be used to monitor and optimise baking conditions with the aim to minimise the shortage of this amino acid. Furosine, formed by the acid hydrolysis of Amadori compounds (stable products of the initial steps of the Maillard reaction), is employed to evaluate heat damage in several food products [11,12]. HMF, generated by the degradation of both sugars and Amadori products [13], is used in bread [14,15], pasta [16], baby cereals [17], etc. GLI, formed by maltose and amino acids, has been proposed as an indicator of a browning reaction in baby cereals and bread [18]. Furfural, from pentose or HMF degradation, is usefully exploited for cookie monitoring during baking [13].

The present work, therefore, aims to: (i) characterize the antioxidant properties (carotenoids, tocols, phenolic acids, antioxidant capacity), protein content and amino acid composition, sugars content and colour of wholemeals of buckwheat, amaranth, quinoa, bread wheat (*Triticum aestivum* L. ssp. *aestivum*) and einkorn wheat (*Triticum monococcum* L. ssp. *monococcum*); and (ii) evaluate the heat damage and colour of biscuits produced using 50% wholemeal or refined einkorn flour plus 50% wholemeal buckwheat, amaranth or quinoa; as controls, water biscuits from 100% wholemeal or refined flour of einkorn and bread wheat were prepared.

## 2. Results and Discussion

The wholemeals of the different species were characterized for selected traits that are relevant in terms of nutritional value and susceptibility to the Maillard reaction upon subsequent processing into water biscuits. The one-way analysis of variance (ANOVA) (Supplementary Table S1) revealed highly significant differences for all the compounds analysed.

### 2.1. Tocols

Presence and levels of tocols were extremely dissimilar among pseudocereals (Table 1). Buckwheat flour showed high concentrations of γ-tocopherol and low of α- and δ-tocopherol. Amaranth contained only α- and β-tocopherol, while quinoa had just α- and γ-tocopherol. For buckwheat, the values were slightly higher than those described in previous studies [19,20], where γ-tocopherol varied between 49.0 and 57.0 mg/kg DM. For amaranth, they were similar to those (15.6–19.1 and 11.4–40.7 mg/kg DM) obtained by different authors [21,22,23]; however, they also detected γ- and δ-tocopherol. Similarly, our quinoa results were in-line with those of other researchers [20,24,25], who reported contents of 8.0–24.0 and 25.8–49.0 mg/kg, respectively; the aforementioned authors found limited quantities of other tocols too.

**Figure 1:**
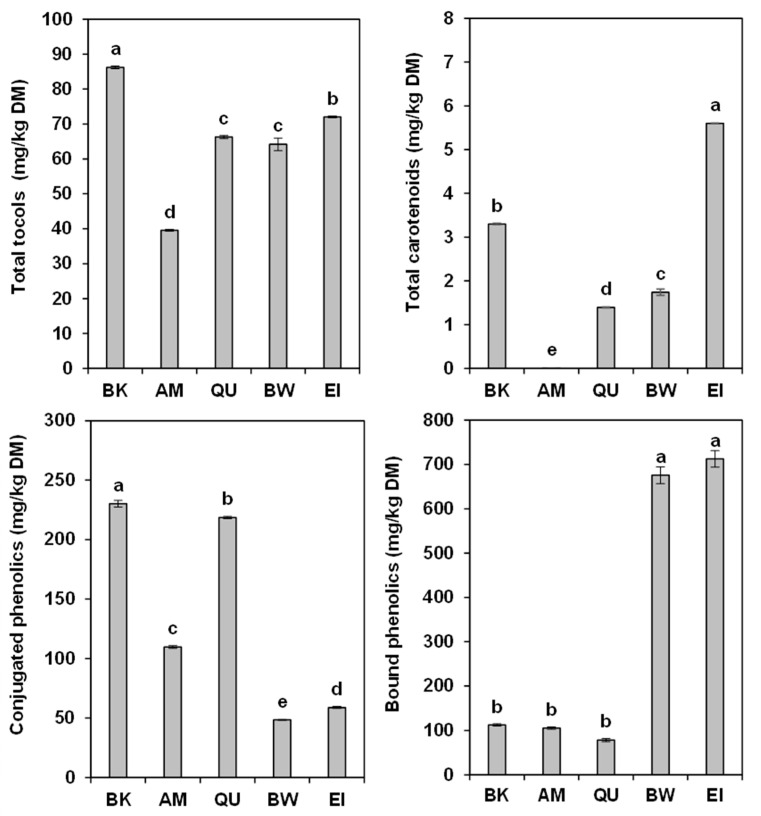
Total tocols, carotenoids, conjugated phenolics and bound phenolics (mg/kg DM) of the buckwheat (BK), amaranth (AM), quinoa (QU), bread wheat (BW) and einkorn (EI) wholemeal. The error bars represent the standard deviation; different letters indicate significant differences (*p* < 0.05) among samples.

The two *Triticum* displayed profiles quite different from those of pseudocereals. Both bread wheat and einkorn contained good concentrations of α- and β-tocopherols, and no γ- and δ-tocopherols; interestingly, they also had remarkable levels of α- and β-tocotrienol. The values of tocols in *Triticum* samples were similar to previous results [26,27,28]. Overall, total tocols content (Figure 1) was maximum in buckwheat (86.2 mg/kg DM), followed by einkorn (72.0 mg/kg DM), quinoa (66.3 mg/kg DM), bread wheat (64.2 mg/kg DM) and amaranth (39.6 mg/kg DM). While bread wheat and einkorn had a more varied composition, the pseudocereals showed high concentrations of specific compounds: in fact, quinoa had the highest content of α-tocopherol, amaranth of β-tocopherol and buckwheat of δ-tocopherol.

### 2.2. Carotenoids

The main carotenoid found in buckwheat (Table 1) was lutein, along with small quantities of zeaxanthin, while quinoa has a limited content of lutein, zeaxanthin and (α+β)-carotene, and amaranth was completely devoid of carotenoids. In general, these results agree with the literature data. Tuan et al. [29] reported slightly higher concentrations of lutein (3.71–6.87 mg/kg DM) and zeaxanthin (0.39–0.50 mg/kg DM) in two buckwheat varieties, plus lower amounts of β-carotene. Instead, our data of lutein and zeaxanthin in quinoa were lower than those (8.2–12.4 and 0.6–0.8 mg/kg DM, respectively) described by Tang et al. [25].

Bread wheat and einkorn showed a greater variety of carotenoids. The former presented lutein and traces of (α+β)-carotene and zeaxanthin. The same carotenoids, with the addition of β-cryptoxanthin, were recovered in einkorn. Their contents were similar to those (3.92–12.64, not detected—2.39, 0.29 and 0.10 mg/kg DM) reported in the literature [27,30,31,32,33,34,35]. Overall (Figure 1), einkorn had the highest concentration of carotenoids (5.60 mg/kg DM), followed by buckwheat (3.30 mg/kg DM), bread wheat (1.74 mg/kg DM) and quinoa (1.40 mg/kg DM). Lutein was by far the most abundant compound in all samples, except amaranth.

### 2.3. Conjugated Phenolic Acids

The conjugated phenolic acids composition of the pseudocereals and of the wheats is presented in Table 1. Buckwheat contained, in decreasing quantities, syringaldehyde, *p*-hydroxybenzoic acid, *p*-coumaric acid and traces of syringic acid. There are not many references of conjugated phenolic acids in buckwheat but these results, apart syringaldehyde, are similar to those reported for *Fagopyrum tataricum*, i.e., 9–34 mg/kg *p*-hydroxybenzoic acid, 1.5–15.9 mg/kg *p*-coumaric acid, 4.9 mg/kg syringic acid, 1.7–26.2 vanillic acid and 1.1–33.5 mg/kg ferulic acid [36].

In amaranth, the conjugated fraction was composed of ferulic acid, *p*-hydroxybenzoic acid, vanillic acid, and coumaric acid (along with traces of syringic acid). These very same phenolic acids were observed in similar quantities (62.1–83.2, 19.7–36.8, 42.8–66.7 and 8.0–9.9 mg/kg) in soluble extracts of four *Amaranthus caudatus* accessions [37]; in addition, caffeic, sinapic and protocatechuic acids were recorded.

Quinoa contained vanillic, ferulic, *p*-coumaric, *p*-hydroxybenzoic and syringic acids in quantities comparable to the results (89.7–146.0, 120–200, 22.6–275.0 and 19.2–38.8 mg/kg, respectively) reported by Repo-Carrasco et al. [37] and to the values (22.84–30.35 mg/kg ferulic acid, 21.56–34.08 mg/kg *p*-coumaric acid, 31.97–51.84 mg/kg *p*-hydroxybenzoic) found by Tang et al. [38].

The conjugated phenolics composition of the two wheats was similar, as both contained ferulic acid, syringic acid, vanillic acid and, in small quantities, *p*-hydroxybenzoic acid, *p*-coumaric acid and syringaldehyde, although einkorn in general showed higher concentrations. These results are in the ranges of those (9.4–62.3, 3.9–22.0, 8.8–24.5, 2.3–11.1, 3.0–12.1 and 0.0–2.9 mg/kg, respectively) reported for bread wheat [39] and (34.1–36.1, 3.5–4.8, 5.1–6.0, 1.6–2.2, 2.2–2.6 and 1.4 mg/kg) for einkorn [40].

### 2.4. Bound Phenolic Acids

Table 1 also depicts the bound phenolic acids composition of all the species tested. Buckwheat contained syringaldehyde, *p*-hydroxybenzoic acid, *p*-coumaric acid, syringic acid, vanillic acid and ferulic acid, in good agreement with previous data (10.0–21.0, 4.8–9.8, 1.1–2.6 and 2.1–5.2 mg/kg for *p*-hydroxybenzoic, ferulic, *p*-coumaric and vanillic acids, but no syringaldehyde) [41] on *Fagopyrum tataricum*. On the other hand, Zhu et al. [42] did not observe vanillic acid, *p*-coumaric acid and syringaldehyde.

Amaranth revealed the presence of ferulic acid (about 80% of total bound phenolic acids), syringic acid, *p*-coumaric acid, *p*-hydroxybenzoic acid and vanillic acid; Okarter [43] identified a similar profile, rich in ferulic acid (45.24 mg/kg DM) and *p*-coumaric acid (11.6 mg/kg DM).

Quinoa had a composition similar to amaranth, rich in ferulic acid (about 80%), and with small quantities of *p*-hydroxybenzoic acid, *p*-coumaric acid, vanillic acid and syringic acid. A likewise composition, but with higher concentrations of all the compounds, i.e., 68.9–89.0, 19.0–21.0, 14.0–21.5 and 14.0–32.3 mg/kg of ferulic, *p*-hydroxybenzoic, *p*-coumaric and vanillic acid acids, was reported [43,44]. The same compounds, with the exception of syringaldehyde and the addition of sinapic acid and protocatechuic acid (but only in coloured quinoa), were also reported [45].

In bread wheat and einkorn wheat, the ferulic acid represented 94–95% of total bound phenolics, followed by *p*-coumaric acid, syringic acid, vanillic acid and *p*-hydroxybenzoic acid, results similar to those described for bread wheat [39] and for einkorn [40].

The total polyphenols content of the five samples was 342.8 (buckwheat), 215.3 (amaranth), 297.1 (quinoa), 724.2 (bread wheat) and 771.5 (einkorn) mg/kg DM. The values of the two wheats were 2.1–3.6 times higher to those of pseudocereals. However, the conjugated phenolics content of buckwheat and quinoa was more than double that of their bound phenolics, while in amaranth the ratio was almost equivalent; a similar finding (0.48 vs. 0.26 mg GAE/g) was reported for quinoa [46]. On the contrary, bread wheat and einkorn showed bound phenolic acids concentrations about 15 times higher than those of the conjugated phenolic acids. In fact, the pseudocereals contained 1.9–4.8 more conjugated phenolics than the two wheats, an interesting result because the conjugated form is of better nutritional value since it is more readily available and can be more easily absorbed by the human body [47].

### 2.5. Antioxidant Capacity

**Figure 2:**
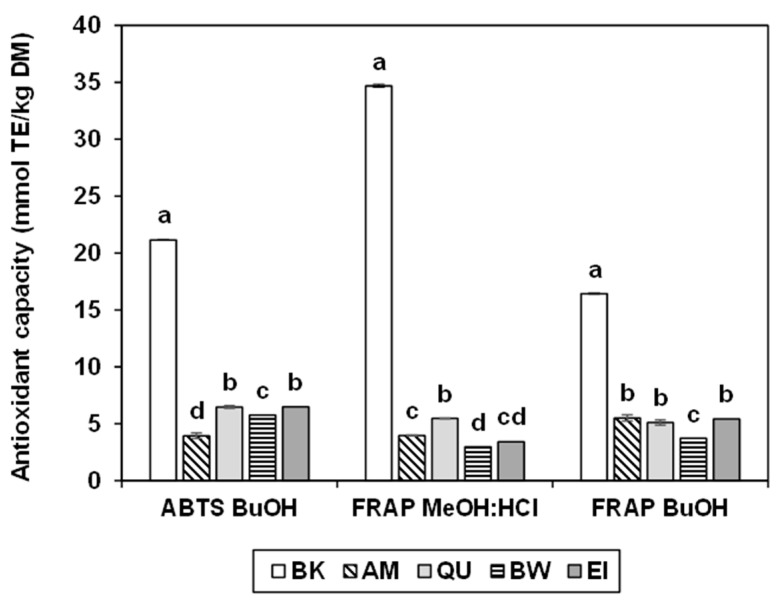
Antioxidant capacity tested by the ABTS and FRAP methods, of the buckwheat (BK), amaranth (AM), quinoa (QU), bread wheat (BW) and einkorn (EI) wholemeal. The error bars represent the standard deviation; different letters indicate significant differences (*p* < 0.05) among samples.

Figure 2 depicts the antioxidant capacity, expressed in mmol Trolox equivalent (TE)/kg DM, of the butanol (BuOH) and acidified methanol (MeOH:HCl) extracts of the wholemeals of pseudocereals and wheats. Buckwheat always showed the greatest antioxidant capacity, with remarkably high values for ABTS_BuOH_ (21.15 mmol TE/kg DM), FRAP_MeOH:HCl_ (34.68 mmol TE/kg DM) and FRAP_BuOH_ (16.44 mmol TE/kg DM). These results are similar to those reported by Lee et al. [48], i.e., 42.5–146 mmol TE/kg DM with ABTS and 14.71–78.2 mmol TE/kg DM with FRAP. The high antioxidant capacity of buckwheat may be due to both the considerable concentration in conjugated polyphenols and to the presence of flavonoids such as rutin, not quantified in our research and absent in other species.

Amaranth, quinoa, bread wheat and einkorn had lower, similar, antioxidant capacity. In amaranth, this varied between 3.94 and 5.50 mmol TE/kg DM, results slightly higher than those (1.96–1.99 mmol TE/kg) described by Gorinstein et al. [49]; in quinoa, this ranged between 5.10 and 6.47 mmol TE/kg DM, i.e., was slightly lower than the 8.5–67.8 mmol TE/kg described for the conjugated fraction [38]; while in bread wheat, this was between 2.97 and 5.76 mmol TE/kg DM and in einkorn between 3.41 and 6.48 mmol TE/kg DM, as already reported by Brandolini et al. [40].

### 2.6. Sugars

**Figure 3:**
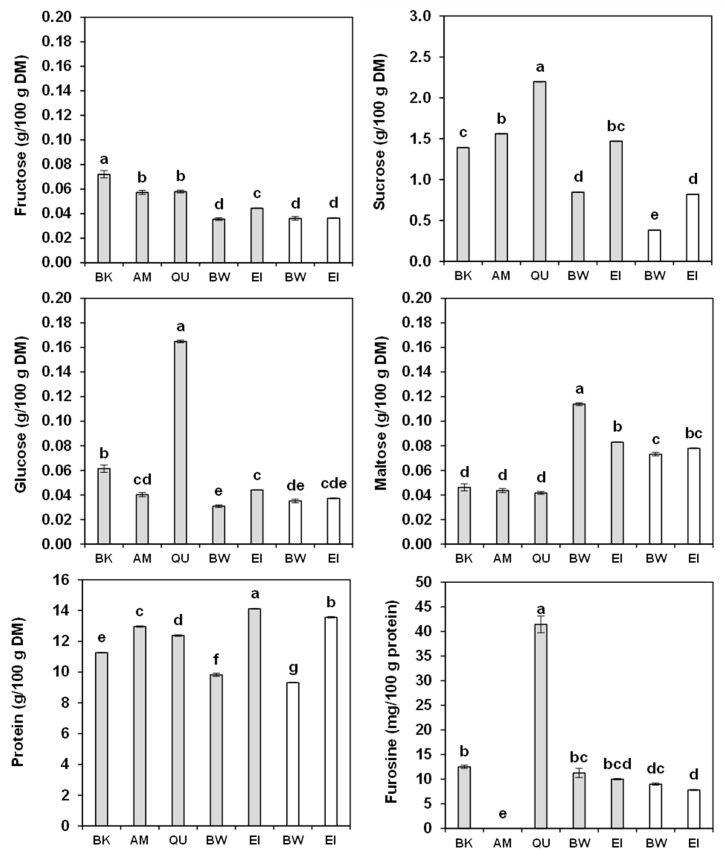
Fructose, glucose, maltose, sucrose, protein (g/100 g DM) and furosine (mg/100 g protein) content of the buckwheat (BK), amaranth (AM), quinoa (QU), bread wheat (BW) and einkorn (EI) wholemeals (grey bars) and of the bread wheat and einkorn refined flours (empty bars). The error bars represent the standard deviation; different letters indicate significant differences (*p* < 0.05) among samples.

The content of reducing sugars (fructose, glucose and maltose) and sucrose of wholemeals and refined flours is shown in Figure 3. The ANOVA (Supplementary Table S2) described the existence of significant differences between samples for all the sugars.

The reducing sugars were scarce, except glucose in quinoa wholemeal (0.16 g/100 g DM) and, to a certain extent, maltose in bread wheat and einkorn wholemeals and refined flours (0.07–0.11 g/100 g DM). Sucrose was scarce in refined flour and wholemeal of bread wheat as well as in refined flour of einkorn (0.80 g/100 g DM); medium in buckwheat (1.39 g/100 g DM), amaranth (1.56 g/100 g DM) and wholemeal einkorn flour (1.47 g/100 g DM); and more abundant in quinoa (2.20 g/100 g DM). In summary, the wheats contained higher amounts of maltose while the pseudocereals contained mainly sucrose and, in the case of quinoa, glucose. Our values corroborate those described for quinoa and amaranth [50], and are similar to those (0.04–0.07, 0.02–0.04, 0.06 and 0.50–0.60 g/100 g for fructose, glucose, maltose and sucrose, respectively) reported for einkorn and bread wheat [51]. Slightly higher quantities of fructose (0.11 g/100 g DM), glucose (1.10 g/100 g DM) and sucrose (2.00 g/100 g DM) in amaranth were sometimes observed [52].

### 2.7. Protein Content and Amino-Acid Composition

It is a well-established fact that the removal of the pericarp and aleuronic layers during milling affects the protein content, as evidenced also by the comparison between wholemeals and refined flours of bread wheat (9.82 vs. 9.31 g/100 g DM) and einkorn (14.12 vs. 13.56 g/100 g) (Figure 3). Nevertheless, einkorn refined flour had a higher protein content than amaranth, quinoa and buckwheat wholemeals (12.96, 12.37 and 11.26 g/100 g, respectively). These results are slightly lower than those reported for bread wheat (12.9–19.9 g/100 g) [53] and for einkorn wheat (14.4–20.7 g/100 g) [54]. Similarly, they are superior to the values recorded for amaranth, quinoa and buckwheat (13.4, 12.2–16.3 and 13.1 g/100 g, respectively) by Mota et al. [55], and for amaranth (15.1–16.4 g/100 g DM) and buckwheat (13.9–16.4 g/100 g DM) by De Bock et al. [56]. However, they are higher than the results reported for amaranth (11.09–12.07 g/100 g DM) found by Repo-Carrasco et al. [37] and are within the variation observed for quinoa (9.5–16.7 and 11.32–14.72 mg/kg DM, respectively) by De Bock et al. [56] and Repo-Carrasco et al. [37].

However, the superior nutritional value of pseudocereal proteins over those of wheats also resides in their balanced composition; they are richer in some essential amino acids, compared to the two wheats (Table 2). The three pseudocereals share a similar amino-acid composition, which differs significantly from that of the two cereals. In fact, compared to the two *Triticum*, buckwheat, amaranth and quinoa are richer in two essential amino acids, threonine and lysine, as well as in aspartate, alanine, glycine, and arginine, with the last two amino acids considered as conditionally essential. Interestingly, in the wholemeal, the pseudocereals lysine content on average is more than double that of the wheats (56.5 vs. 25.5 g/kg protein, respectively). The content of the essential branched-chain amino acids, namely, leucine, isoleucine, and valine, is also remarkable in pseudocereals; conversely, they are low in glutamate and proline. However, it should be underlined that the acid hydrolysis required for analysing the amino-acidic composition of proteins de facto prevents the determination of some amino acids. This is the case of glutamine and asparagine, which upon hydrolysis are deaminated into glutamate and aspartate, respectively, and tryptophan, which is unstable to acidic conditions and requires alkaline hydrolysis [57]. Overall our results confirm previous reports on buckwheat [58,59], amaranth [60,61,62], quinoa [62,63,64], bread wheat and einkorn [65,66]. Differences merely concerm the content of methionine and tyrosine, which are both sensitive to oxidation and, thus, critical to analyse.

### 2.8. Furosine

The furosine levels of the wholemeals and refined flours tested are presented in Figure 3. Furosine is a derivative of the Amadori compounds formed from reducing sugars and the ε-amino group of lysine during the initial stages of the Maillard reaction. Thus furosine may represent a useful indicator of heat damage endured by wholemeals and refined flours during the milling process. Amaranth did not show any detectable furosine, while buckwheat, bread wheat and einkorn presented minimal levels (7.8–12.5 mg/100 g protein), in line with the results (3.5–14.8 mg/100 g protein) reported by Hidalgo and Brandolini [51] for refined flours of different wheats. Quinoa, on the other hand, had a greater furosine concentration (41.4 mg/100 g protein), likely as a consequence of its higher glucose content. Additionally, our quinoa was purchased at the market and its post-harvest treatments are unknown: it is possible that the seeds were heat-dried, after the preliminary washing to eliminate endogenous saponins, thus inducing heat damage.

### 2.9. Water Biscuits Heat Damage

**Figure 4:**
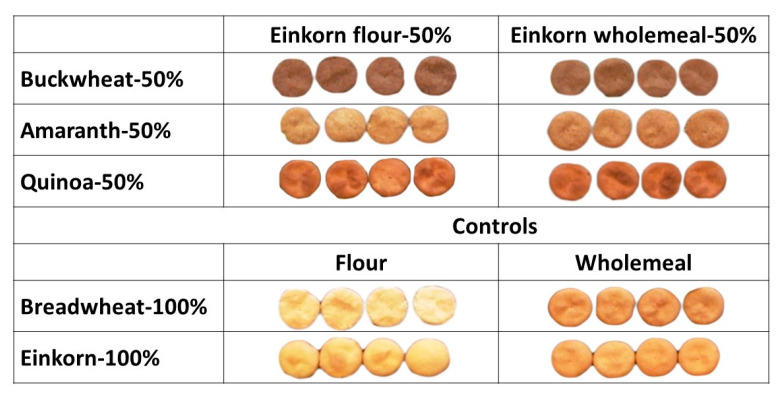
Water biscuits prepared with 50% einkorn flour and 50% buckwheat, amaranth, or quinoa wholemeal, or 100% bread wheat flour or 100% einkorn flour (on the **left**) and water biscuits prepared with 50% einkorn wholemeal and 50% buckwheat, amaranth, or quinoa wholemeal, or 100% bread wheat wholemeal or 100% einkorn wholemeal (on the **right**).

Figure 4 depicts the different water biscuits and their formulations.

**Figure 5:**
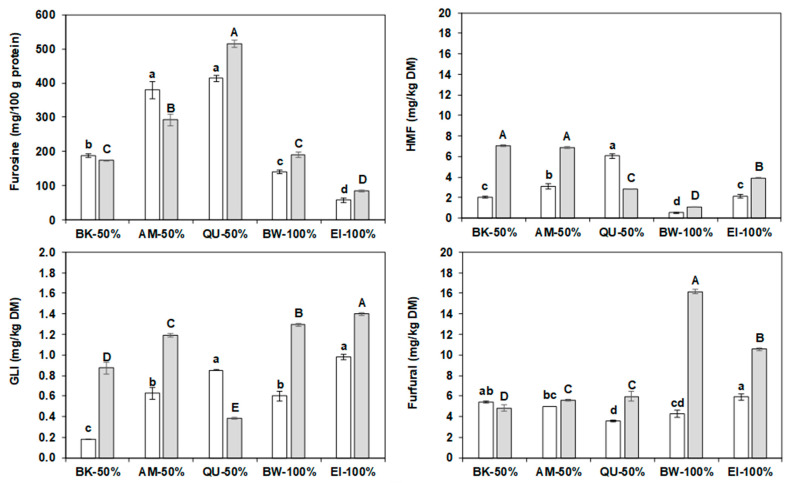
Furosine (mg/kg protein), hydroxymethylfurfural (HMF), glucosylisomaltol (GLI), and furfural (mg/kg DM) levels of the water biscuits. Empty bars: water biscuits prepared with 50% einkorn flour and 50% buckwheat (BK), amaranth (AM), or quinoa (QU) wholemeal, or 100% bread wheat (BW) flour or 100% einkorn (EI) flour. Grey bars: water biscuits prepared with 50% einkorn wholemeal and 50% buckwheat, amaranth, or quinoa wholemeal, or 100% bread wheat wholemeal or 100% einkorn wholemeal. The error bars represent the standard deviation; different lower-case letters indicate significant differences (*p* < 0.05) among samples prepared with einkorn refined flour, while capital letters indicate significant differences (*p* < 0.05) among samples prepared with einkorn wholemeal.

Supplementary Table S3 reports the two-way ANOVA of heat-damage (furosine, GLI, HMF and furfural) and colour-coordinates results of the water biscuits (WB) obtained from wholemeal flours or refined flours of bread wheat, einkorn and einkorn mixtures with pseudo-cereal flours. The ANOVA showed significant differences (*p* < 0.05) for both the type of flour (refined or wholemeal) and the type of wheat (bread wheat or einkorn or blend). Figure 5 shows the results for the heat-damage evaluation in different WB. The furosine values were barely different (*p* ≤ 0.046) between WB containing only wholemeal flours and from WB with refined flour, except for those with 50% amaranth and quinoa. The control WB, obtained only from bread wheat or einkorn, had the lowest values, both for refined and wholemeal samples.

The GLI content varied depending on the type of flour but also on the pseudocereals. This compound reached much higher values in WB with einkorn wholemeal than in WB with einkorn refined flour, apart from those with 50% quinoa. The HMF content showed the advancement of heat damage but this was lower in the samples with refined flour than in those with only wholemeal, again with the exception of those containing quinoa. The WB obtained from the mixtures with buckwheat and amaranth reached significantly higher values, especially if integral, compared to the controls. The furfural content, which underscores the further advancement of heat damage, was lower in the WB from refined wheats flours compared to those from wholemeal wheat flours (on average 5.0 vs. 8.5 mg/kg DM), except in the buckwheat-enriched samples. The control WB from wholemeal presented furfural concentrations far higher than all the other WB.

Overall, the WB from wholemeal flours suffered greater heat damage than those obtained from refined flour, regardless the formulation, in agreement with the higher protein content of the former. Indeed, protein usually represents the limiting reagent for the Maillard reaction in cereal products [16]. Furthermore, the control WB seemed to have suffered more heat damage than those obtained from mixtures enriched with pseudocereals, since they had lower furosine but higher GLI (1.1 vs. 0.7 mg/kg DM) and furfural (9.2 vs. 5.9 mg/kg DM) values, clearly indicating the advanced changes undergone by the products of the Maillard reaction.

Comparing the levels of furosine in WB with the literature data is difficult because very few articles report about comparable products (i.e., with no sugar and no lipid addition). In general, the control WB from refined flour had a furosine level close to the values (16.5–81.5 mg/100 g protein) reported by Hidalgo and Brandolini [67] for WB of three wheat species baked for 25 min. The levels in WB enriched with pseudocereals showed a trend (quinoa > amaranth > buckwheat > bread wheat > einkorn) similar to that observed by Hidalgo et al. [68,69]. The GLI of the WB with refined flour was in the lower end of the range (n.d.—11.4 mg/kg DM) observed by Hidalgo and Brandolini [67]; however, unlike our results, their HMF and furfural were always below the detection limit. Interestingly, an HMF content in the WB with pseudocereals greater than in the wheats WB was reported [68,69].

Considering the amino-acid profile of water biscuits before (theoretical) and after baking (Table 2), a slight degradation was observed for almost all these compounds, particularly in *Triticum* samples not enriched with pseudocereals, because of the harsh processing conditions. Beside this, lysine and arginine contents were lower in baked WB due to the direct involvement of these amino acids in the Maillard reaction, regardless the type of WB formulation. In contrast, the decreased contents of cysteine, methionine and tyrosine were likely the result of oxidation phenomena occurring during baking [70]. However, despite the observed losses, the amino-acid profile remained more balanced in WB fortified with pseudocereals.

### 2.10. Colour of Flours and Water Biscuits

**Figure 6:**
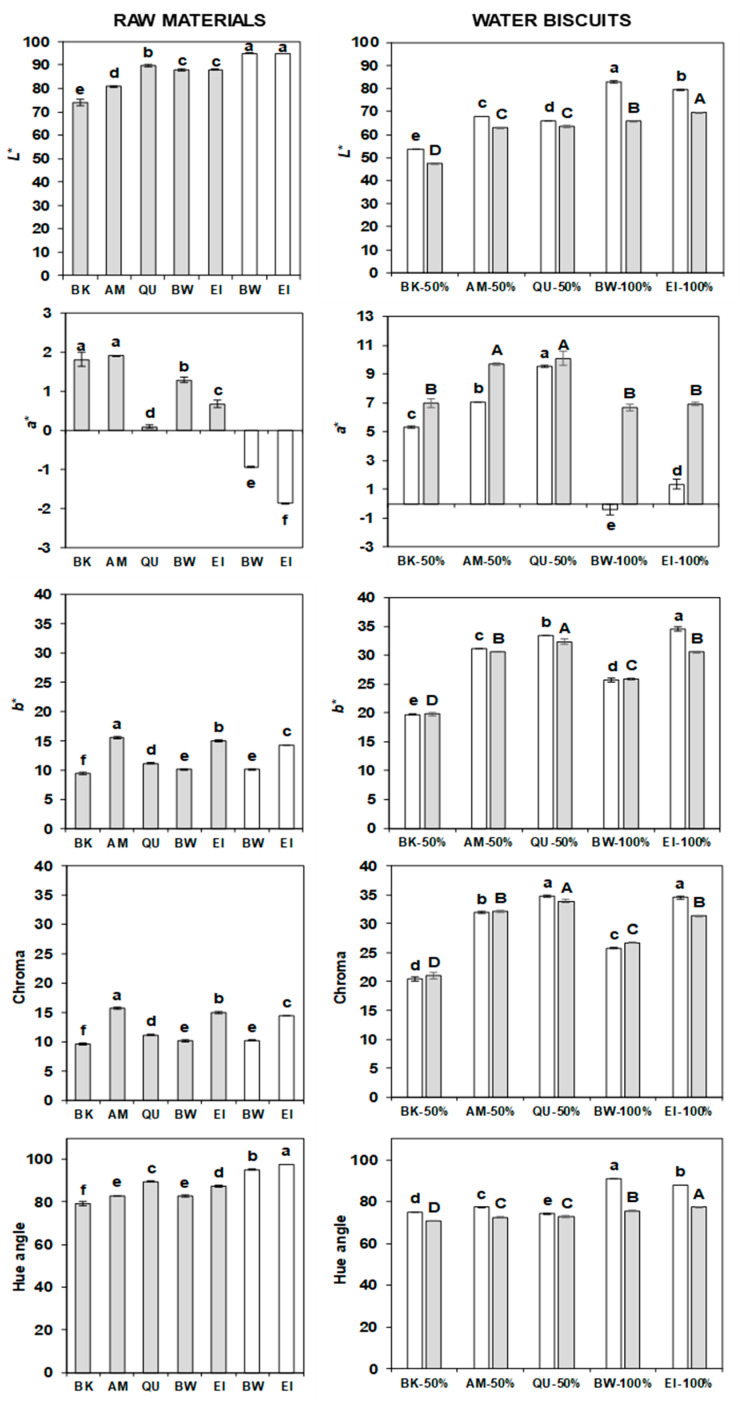
Colour coordinates, chroma and hue angle of the buckwheat, amaranth, quinoa, bread wheat and einkorn wholemeal and of the bread wheat ad einkorn refined flours (**left** side) and of the water biscuits prepared with 50% einkorn flour and 50% amaranth, quinoa or buckwheat wholemeal, or 100% einkorn flour or 100% bread wheat flour (empty bars) or prepared with 50% einkorn wholemeal and 50% amaranth, quinoa or buckwheat wholemeal, or 100% einkorn wholemeal or 100% bread wheat wholemeal (grey bars). The error bars represent the standard deviation; different lower case letters indicate significant differences (*p* < 0.05) among samples prepared with einkorn refined flour, while capital letters indicate significant differences (*p* < 0.05) among samples prepared with einkorn wholemeal (**right** side).

The ANOVA highlighted the existence of significant differences among wholemeals/flours (Supplementary Table S2), and among water biscuits (Supplementary Table S3). The values of the three colour coordinates L* (brightness), a* (red-green) and b* (yellow-blue), as well as Chroma and Hue angle, are depicted in Figure 6.

The L* parameter was smallest in the wholemeal of buckwheat (73.9), characterised by a greyish flour; slightly higher in that of amaranth (80.7); higher in those of quinoa (89.7), bread wheat and einkorn (88.0 in both); and much higher in the refined flours of wheat and einkorn (94.0 in both). The a* component was always low, ranging between −1.87 (refined einkorn) and 1.92 (amaranth), but the refined flours had negative values while the wholemeals had positive values. Finally, b* was low in wholemeal of buckwheat, quinoa, bread wheat and einkorn (9.5, 11.2, 10.1 and 15.0, respectively) and higher in refined amaranth, bread wheat and einkorn (15.6, 10.2 and 14.3, respectively). Overall, refined flours were lighter than wholemeals due to the removal of the dark outer layers of the kernels.

The trend in the flours was noticed also in the WB: those enriched with buckwheat wholemeal were darkest, followed by the quinoa and amaranth-enriched WB and the controls; Hidalgo et al. [68] observed the same situation. Similarly, the samples with refined flour were brighter and had lower a* than those with only wholemeal, while the differences in b* were mainly species-specific. The colour coordinates were not strictly related to the heat damage, because they were influenced by the seeds’ colour: for example, the WB with buckwheat wholemeal had the lowest luminosity (53.5 vs. the average 69.2 of all the others) and b* (21.2 vs. 30.9) but showed also low heat-damage values.

In the flours, the Chroma and the Hue angle ranged from 9.7 to 15.7 and from 79.2° to 97.4° (approximately corresponding to the yellow region, i.e., 90°), respectively. The highest Chroma values were observed in amaranth wholemeal and einkorn wholemeal and flour. After baking, the saturation (Chroma) increased. Minimal differences existed between the pseudocereals-enriched WB prepared from einkorn flour and from einkorn wholemeal. The quinoa-enriched WB showed the highest Chroma, followed by the 100% einkorn, amaranth-enriched, 100% bread wheat and buckwheat-enriched WB. The refined flours had the highest Hue scores, and the buckwheat wholemeal exhibited the lowest values. After baking, the Hue decreased towards a redder region: 74.1–90.9° in the pseudocereals-enriched WB from einkorn flour and 70.6–77.2° in the enriched WB from einkorn wholemeal. Significant, but small, differences were noticed among the pseudocereals-enriched WB prepared from einkorn wholemeal. All the changes in Chroma and Hue observed after baking are attributable to the formation of brownish compounds [71].

## 3. Materials and Methods

### 3.1. Materials

Amaranth (*Amaranthus cruentus* L.) cv. MT-3, buckwheat Italian local population Seis, einkorn cv. Monlis and biscuit-type bread wheat cv. Bramante were cropped in the Sant’Angelo Lodigiano (LO) fields of CREA-ZA, while the quinoa seeds were purchased from the commercial circuit.

### 3.2. Methods

#### 3.2.1. Wholemeal and Refined Flour Preparation

After harvesting, the seeds were stored at 5 °C. Immediately prior to milling, the kernels of Monlis were de-hulled with an Otake FC4S thresher (Satake, Hiroshima, Japan). The wholemeal flours of the two wheats and three pseudocereals were prepared using a Cyclotec 1093 laboratory mill (FOSS Tecator, Hillerød, Denmark), while the refined flours of bread wheat and einkorn wheat were made using a Bona 4RB (Bona, Monza, Italy) experimental mill, which separates the flour fraction from bran and germ.

#### 3.2.2. Water Biscuits Production

A flowchart of water-biscuit preparation and analyses is presented in Supplementary Figure S1. Ten different water-biscuit types were prepared: five were made with 50% einkorn flour and 50% amaranth, quinoa or buckwheat wholemeal, or 100% einkorn flour or 100% bread wheat flour, while five were prepared with 50% einkorn wholemeal and 50% amaranth, quinoa or buckwheat wholemeal, or 100% einkorn wholemeal or 100% bread wheat wholemeal. The WB were prepared using only deionized water and flour, to unambiguously determine flour role in the Maillard reaction. In particular, for each type of biscuit, 80 g of flour (or combinations of flours) at 14% moisture and 35 mL of water were mixed for 90 s using a Hobart C-100 electric mixer (National MFG Co., Lincoln, NE, USA). Subsequently, the dough was rolled to a 7 mm homogeneous sheet and cut with a die-cutter (internal diameter of 60 mm) to obtain two WB, and baked in an Ovenlab rotary oven (National MFG Co., Lincoln, NE, USA) at 205 °C for 30 min. After cooling at room temperature for 30 min, the WB were stored at −20 °C. Just before analysis, they were ground with a laboratory mill (Braun, Waiblingen, Germany). Two batches of each WB type were prepared.

### 3.3. Analyses

The following analyses were performed on the wholemeals: dry matter (DM), content of sugars, proteins, tocols, carotenoids, soluble conjugated phenolic acids, bound insoluble phenolic acids, antioxidant capacity, amino-acids composition, furosine and colour. The water biscuits were characterised for dry matter, protein, amino-acids composition, the heat damage parameters furosine, GLI, HMF and furfural, and colour.

The dry matter was determined gravimetrically according to the official method 44–15A [72]. Fructose, glucose, maltose and sucrose were investigated by HPLC [51]. The protein content was computed multiplying by the factor 5.70 the total nitrogen content determined with the Kjeldahl 46–10 method [72]. Tocols, carotenoids [73], soluble conjugated and bound insoluble phenolic acids [74] were extracted and quantified by HPLC. Antioxidant capacity of the butanol extracts was evaluated by the ABTS [75] and FRAP [76] methods, while acidified methanol extracts (80:20, methanol:HCl 1%) were assessed only by FRAP because the very acidic pH of the extract rapidly discoloured the ABTS solution, making the analysis impossible. The extractions were performed as described by Yilmaz et al. [74]. Furosine, HMF, GLI and furfural were determined by HPLC [51].

Amino-acid composition was assessed following the method described by Hogenboom et al. [77]. Briefly: the hydrolysed samples were purified by solid-phase extraction on a C18 cartridge previously activated with 5 mL of methanol and 10 mL of water. One mL of filtrate was transferred to a 25 mL flask and added with approximately 20 mL of 0.2 M lithium citrate buffer at pH 2.20. The pH was adjusted to 2.20 and the volume was made up with the lithium citrate buffer. Before injection into the chromatograph, the sample was filtered on an 0.2 µm RC filter. Amino-acids separation was performed by ion exchange chromatography (IEC) using a Biochrom 30+ Amino Acid Analyzer (Biochrom Ltd., Cambridge, UK) chromatograph, under the conditions indicated by the manufacturer. Detection was performed at 570 nm for the primary amino acids (AA) and at 440 nm for the secondary ones (proline), after post-column derivatization with ninhydrin. The separation of AA was achieved by injecting 100 µL of sample in an ion exchange column (120 mm × 4.6 mm) packed with a sulphonic resin supplied by Biochrom. For the quantification of AA, 4-point calibration lines of the reference standards were used.

Colour, measured with a reflectance Minolta Chroma meter II colorimeter (Minolta Italia SpA, Milan, Italy), was expressed using the CIE L*, a*, b* coordinates system, where L* is lightness, a* is redness and b* is yellowness. Chroma and Hue angle were calculated considering the equations [78]: $Chroma=\sqrt{a^{*2}+b^{*2}}$; $Hue\;angle=arctan\;\left(b^{*}/a^{*}\right)$.

All analytical measurements were performed twice; the results are presented as means (±standard deviation).

### 3.4. Statistical Analysis

To evaluate the influence of the species and their mixtures on composition, heat damage and colour of wholemeals, refined flours, and WB, the data were processed by one-way analysis of variance (ANOVA). The WB data were also evaluated by two-way ANOVA, considering the type of flour (refined or wholemeal) and the blend as factors. When significant differences (*p* < 0.05) were found, Fisher’s lowest significant difference (LSD) at a significance level of 95% was determined. ANOVA and LSD test were performed with the Centurion XVI statistical package (Statgraphics Technologies, Inc., The Plains, VA, USA).

## 4. Conclusions

The analysis of wholemeal flours showed that all the tested pseudocereals were particularly rich in soluble conjugated phenolic acids, while the wheats contained mainly insoluble bound phenolic acids. Buckwheat had the highest content of total tocols and einkorn the highest content of total carotenoids. While the protein content did not differ much between species, the pseudocereals confirm a more balanced amino-acid profile, characterized by good levels of lysine. All the WB obtained using einkorn wholemeal suffered greater heat damage than those obtained from einkorn refined flour, regardless the presence of a pseudocereal. However, the control WB produced with bread wheat and einkorn showed greater heat damage than those enriched with pseudocereals. The colour coordinates were mostly influenced by the characteristic colour of the seeds of each species. The significant content of antioxidant compounds, the better amino-acid composition and the lower susceptibility to heat damage of the WB enriched with pseudocereals support a better nutritional value of such bakery products.

## Figures and Tables

**Table 1:** Carotenoid, tocol and phenolic composition (mg/kg DM) of wholemeals of buckwheat, amaranth, quinoa, bread wheat and einkorn wheat.

	Buck Wheat	Amaranth	Quinoa	Bread Wheat	Einkorn Wheat
*Tocols*					
α-tocopherol	3.50 ^d^ ± 0.10	10.41 ^c^ ± 0.04	30.18 ^a^ ± 0.44	12.66 ^b^ ± 0.04	13.13 ^b^ ± 0.15
β-tocopherol	nd ^d^	29.16 ^a^ ± 0.28	nd ^d^	9.15 ^b^ ± 0.41	5.70 ^c^ ± 0.05
γ-tocopherol	77.63 ^a^ ± 0.24	nd ^c^	36.11 ^b^ ± 0.01	nd ^c^	nd ^c^
δ-tocopherol	4.89 ^a^ ± 0.05	nd ^b^	nd ^b^	nd ^b^	nd ^b^
α-tocotrienol	nd ^c^	nd ^c^	nd ^c^	4.15 ^b^ ± 0.08	12.38 ^a^ ± 0.04
β-tocotrienol	nd ^c^	nd ^c^	nd ^c^	38.22 ^b^ ± 1.27	40.81 ^a^ ± 0.02
*Carotenoids*					
(α+β)-carotene	nd ^c^	nd ^c^	0.15 ^b^ ± 0.00	0.16 ^b^ ± 0.01	0.44 ^a^ ± 0.01
Β-cryptoxanthin	nd ^b^	nd ^b^	nd ^b^	nd ^b^	0.04 ^a^ ± 0.00
Lutein	3.17 ^b^ ± 0.01	nd ^e^	1.18 ^d^ ± 0.00	1.43 ^c^ ± 0.05	4.80 ^a^ ± 0.01
Zeaxanthin	0.13 ^b^ ± 0.01	nd ^d^	0.06 ^c^ ± 0.01	0.15 ^b^ ± 0.01	0.33 ^a^ ± 0.01
*Conjugated phenolics*					
*p*-hydroxybenzoic acid	53.72 ^a^ ± 1.18	43.30 ^b^ ± 0.09	32.90 ^c^ ± 0.08	2.58 ^d^ ± 0.02	2.51 ^d^ ± 0.17
Vanillic acid	nd ^d^	6.52 ^c^ ± 0.18	83.42 ^a^ ± 1.14	7.79 ^bc^ ± 0.03	8.45 ^b^ ± 0.14
Syringic acid	0.12 ^c^ ± 0.00	0.12 ^c^ ± 0.00	3.03 ^b^ ± 0.01	6.62 ^a^ ± 0.26	6.26 ^a^ ± 0.33
Syringaldehyde	165.40 ^a^ ± 2.13	nd ^c^	nd ^c^	1.09 ^b^ ± 0.03	2.26 ^b^ ± 0.11
*p*-coumaric acid	10.95 ^b^ ± 0.49	5.41 ^c^ ± 0.10	39.47 ^a^ ± 0.11	1.28 ^e^ ± 0.03	2.98 ^d^ ± 0.08
Ferulic acid	nd ^e^	54.30 ^a^ ± 0.69	59.76 ^a^ ± 0.26	29.13 ^d^ ± 0.60	36.49 ^c^ ± 0.97
*Bound phenolics*					
*p*-hydroxybenzoic acid	24.46 ^a^ ± 0.18	6.56 ^b^ ± 0.25	5.36 ^c^ ± 0.36	2.44 ^d^ ± 0.04	3.33 ^d^ ± 0.48
Vanillic acid	7.80 ^a^ ± 0.07	1.19 ^e^ ± 0.05	3.60 ^d^ ± 0.02	6.80 ^b^ ± 0.20	4.50 ^c^ ± 0.07
Syringic acid	8.61 ^a^ ± 0.15	3.99 ^c^ ± 0.37	2.86 ^d^ ± 0.05	8.66 ^a^ ± 0.15	5.87 ^b^ ± 0.30
Syringaldehyde	43.04 ^a^ ± 0.68	nd ^b^	nd ^b^	nd ^b^	nd ^b^
*p*-coumaric acid	17.10 ^b^ ± 0.99	9.29 ^c^ ± 0.22	3.82 ^d^ ± 0.20	16.57 ^b^ ± 0.32	33.30 ^a^ ± 1.01
Ferulic acid	11.62 ^c^ ± 0.65	84.60 ^b^ ± 2.90	62.88 ^b^ ± 2.93	641.25 ^a^ ± 18.11	665.51 ^a^ ± 18.25

Different letters in the same row highlight significant differences (*p* ≤ 0.05) among samples following Duncan’s LSD test.

**Table 2:** Amino-acid composition (g/kg protein) of wholemeals, refined flours, and water biscuits before (theoretical) and after baking. BK, buckwheat; AM, amaranth; QU, quinoa; BW, bread wheat cv. Bramante; EI, einkorn wheat cv. Monlis.

	Asp	Thr	Ser	Glu	Gly	Ala	Val	Cys	Met	Ile	Leu	Tyr	Phe	Lys	His	Arg	Pro	Total
Wholemeal																		
BK	92.9 ± 1.5	37.5 ± 0.4	45.4 ± 0.2	170.9 ± 0.3	58.9 ± 0.2	41.6 ± 0.3	51.2 ± 0.0	14.9 ± 0.3	2.4 ± 0.3	38.2 ± 0.1	64.9 ± 0.2	13.2 ± 0.3	47.1 ± 0.6	56.9 ± 0.1	23.3 ± 0.2	98.6 ± 0.3	37.2 ± 0.1	895.9 ± 2.6
AM	84.7 ± 0.0	36.6 ± 0.2	56.2 ± 0.2	169.5 ± 1.5	78.9 ± 0.1	36.5 ± 0.2	42.7 ± 0.5	15.8 ± 0.2	10.0 ± 0.3	39.3 ± 0.1	56.6 ± 0.0	17.1 ± 0.4	43.3 ± 0.4	58.0 ± 0.6	25.1 ± 0.5	97.1 ± 0.1	38.1 ± 0.7	905.9 ± 0.1
QU	83.1 ± 0.4	34.5 ± 0.4	39.8 ± 0.1	142.1 ± 0.9	54.6 ± 0.2	41.2 ± 0.4	47.2 ± 0.1	6.9 ± 0.8	5.0 ± 0.2	40.5 ± 0.2	63.8 ± 0.1	14.3 ± 0.4	42.1 ± 0.1	54.7 ± 0.1	26.9 ± 0.2	92.4 ± 0.7	36.3 ± 0.3	825.8 ± 3.7
BW	52.3 ± 0.0	28.1 ± 0.3	42.6 ± 0.2	288.3 ± 0.1	41.0 ± 0.1	33.6 ± 0.1	43.0 ± 0.6	12.7 ± 0.2	4.9 ± 0.1	34.4 ± 0.2	65.2 ± 0.2	11.6 ± 0.7	45.9 ± 0.6	27.3 ± 0.2	21.1 ± 0.6	48.7 ± 0.0	96.1 ± 0.3	897.1 ± 2.7
EI	47.1 ± 0.3	24.7 ± 0.0	41.5 ± 0.3	277.9 ± 0.9	31.6 ± 0.2	29.0 ± 0.0	40.7 ± 0.8	14.2 ± 0.1	8.3 ± 0.2	37.5 ± 0.4	61.3 ± 0.0	11.3 ± 0.5	45.0 ± 0.4	23.8 ± 0.2	20.2 ± 0.0	43.6 ± 0.0	86.0 ± 0.0	843.9 ± 2.3
									Flour									
BW	46.5 ± 0.5	27.0 ± 0.7	45.8 ± 0.7	347.2 ± 3.1	39.0 ± 0.3	31.3 ± 0.3	44.2 ± 0.2	10.4 ± 0.5	9.4 ± 0.8	37.9 ± 0.8	70.2 ± 1.1	10.6 ± 0.2	50.1 ± 1.1	23.5 ± 0.2	21.3 ± 0.3	40.8 ± 2.1	117.4 ± 1.8	972.9 ± 9.7
EI	42.4 ± 1.3	25.7 ± 0.1	47.8 ± 0.4	353.5 ± 0.5	30.1 ± 0.1	27.3 ± 0.2	43.8 ± 0.0	19.1 ± 0.5	11.6 ± 0.3	44.4 ± 0.0	70.1 ± 0.4	11.7 ± 0.3	54.0 ± 0.2	21.3 ± 0.6	21.3 ± 0.2	40.3 ± 0.1	108.9 ± 0.2	973.3 ± 1.0
Water biscuits (before baking)																		
BK 50%	70.8 ± 0.5	33.0 ± 0.3	49.7 ± 0.4	288.8 ± 2.3	47.0 ± 0.4	36.0 ± 0.4	50.4 ± 0.4	18.1 ± 0.3	8.2 ± 0.1	44.5 ± 0.7	72.7 ± 1.1	13.4 ± 0.6	54.3 ± 0.5	40.5 ± 0.3	23.7 ± 0.3	71.5 ± 1.9	81.6 ± 1.2	1007 ± 6.1
AM 50%	62.7 ± 0.5	30.6 ± 0.2	51.3 ± 0.5	266.1 ± 2.1	52.8 ± 0.5	31.4 ± 0.3	43.1 ± 0.3	17.2 ± 0.2	10.9 ± 0.2	41.8 ± 0.6	63.7 ± 1.0	14.2 ± 0.6	48.7 ± 0.5	38.6 ± 0.3	22.9 ± 0.3	66.7 ± 1.7	75.6 ± 1.1	938.2 ± 5.6
QU 50%	67.1 ± 0.5	31.9 ± 0.3	46.5 ± 0.4	265.7 ± 2.1	44.8 ± 0.4	36.2 ± 0.4	48.4 ± 0.4	13.7 ± 0.2	8.4 ± 0.1	45.2 ± 0.7	71.6 ± 1.1	13.9 ± 0.6	51.1 ± 0.5	40.5 ± 0.3	25.5 ± 0.3	70.3 ± 1.8	78.1 ± 1.1	958.9 ± 5.8
BW	46.5 ± 0.5	27.0 ± 0.7	45.8 ± 0.7	347.2 ± 3.1	39.4 ± 0.3	31.3 ± 0.3	44.2 ± 0.2	10.4 ± 0.5	9.4 ± 0.8	37.9 ± 0.8	70.2 ± 1.1	10.6 ± 0.2	50.1 ± 1.1	23.5 ± 0.2	21.3 ± 0.3	40.8 ± 2.1	117.4 ± 1.8	972.9 ± 9.7
EI	42.4 ± 1.3	25.7 ± 0.1	47.8 ± 0.4	353.5 ± 0.5	30.1 ± 0.1	27.3 ± 0.2	43.8 ± 0.0	19.1 ± 0.5	11.6 ± 0.3	44.4 ± 0.0	70.1 ± 0.4	11.7 ± 0.3	54.0 ± 0.2	21.3 ± 0.6	21.3 ± 0.2	40.3 ± 0.1	108.9 ± 0.2	973.3 ± 1.0
Water biscuit (after baking)																		
BK 50%	66.2 ± 0.2	31.7 ± 0.0	48.8 ± 0.4	291.4 ± 0.1	43.8 ± 0.0	34.6 ± 0.2	49.0 ± 0.5	13.0 ± 0.0	4.8 ± 0.0	42.9 ± 0.0	70.0 ± 0.2	10.3 ± 0.1	51.1 ± 0.0	30.8 ± 0.1	22.4 ± 0.4	58.9 ± 0.1	83.6 ± 0.4	953.9 ± 0.2
AM 50%	62.2 ± 0.1	30.8 ± 0.1	51.8 ± 0.2	270.8 ± 0.4	52.8 ± 0.2	31.8 ± 0.1	44.0 ± 0.6	14.7 ± 0.1	9.9 ± 0.2	42.2 ± 0.3	64.0 ± 0.1	10.5 ± 0.9	48.1 ± 0.8	28.5 ± 0.2	23.4 ± 0.2	59.4 ± 0.1	77.6 ± 0.3	923.0 ± 0.2
QU 50%	63.1 ± 0.4	30.3 ± 0.0	43.9 ± 0.5	259.2 ± 0.0	44.1 ± 0.0	36.2 ± 0.1	47.5 ± 0.6	12.7 ± 0.2	7.4 ± 0.0	44.1 ± 0.2	68.2 ± 0.1	10.9 ± 0.2	47.1 ± 0.4	24.7 ± 0.1	23.9 ± 0.2	61.0 ± 0.1	80.7 ± 0.1	905.7 ± 2.3
BW	41.0 ± 0.2	24.5 ± 0.3	41.9 ± 0.4	319.3 ± 0.5	35.5 ± 0.1	28.8 ± 0.1	40.9 ± 0.2	14.7 ± 0.2	9.5 ± 0.2	35.4 ± 0.2	64.2 ± 0.2	10.9 ± 0.3	46.2 ± 0.2	19.6 ± 0.1	19.9 ± 0.2	39.2 ± 0.5	109.8 ± 0.9	901.4 ± 0.7
EI	39.1 ± 0.1	22.8 ± 0.2	42.4 ± 0.3	316.3 ± 0.1	27.6 ± 0.1	25.2 ± 0.1	40.4 ± 0.3	15.8 ± 0.0	10.4 ± 0.1	40.2 ± 0.1	62.8 ± 0.2	9.6 ± 0.2	48.2 ± 0.1	17.4 ± 0.0	18.9 ± 0.2	35.3 ± 0.1	98.0 ± 0.3	870.8 ± 1.3

## Data Availability

All the data related to this work are given here in the manuscript.

## Supplementary Materials

The following supporting information can be downloaded at: https://www.mdpi.com/article/10.3390/molecules27217541/s1, Table S1: Analysis of variance (ANOVA) of antioxidants content and antioxidant capacity of the wholemeals of buckwheat, amaranth, quinoa, bread wheat and einkorn (***, *p* ≤ 0.001); Table S2: Analysis of variance (ANOVA) of sugars, ash, protein and furosine content, and colour coordinates of the wholemeals of buckwheat, amaranth, quinoa, bread wheat and einkorn (***, *p* ≤ 0.001); Table S3: Analysis of variance (ANOVA) of the heat-damage indices and colour coordinates in water biscuits from wholemeal or refined flour of einkorn or bread wheat and from 50:50 blends of einkorn wholemeal or refined flour and pseudocereals wholemeal (* *p* ≤ 0.05, *** *p* ≤ 0.001); Figure S1: Research flowchart.

## Sample Availability

Samples of the compounds are not available from the authors.
