# Supplementary material for: Antioxidants of Amaranth, Quinoa and Buckwheat Wholemeals and Heat-Damage Development in Pseudocereal-Enriched Einkorn Water Biscuits
Source: Molecules. 2022 Nov 3;27(21):7541. doi: 10.3390/molecules27217541 (PMC9654256; doi:10.3390/molecules27217541)
Supplement: Supplementary file 1 [file molecules-27-07541-s001.zip › molecules-1998637-supplementary.pdf]

Figure S1. Research flowchart.

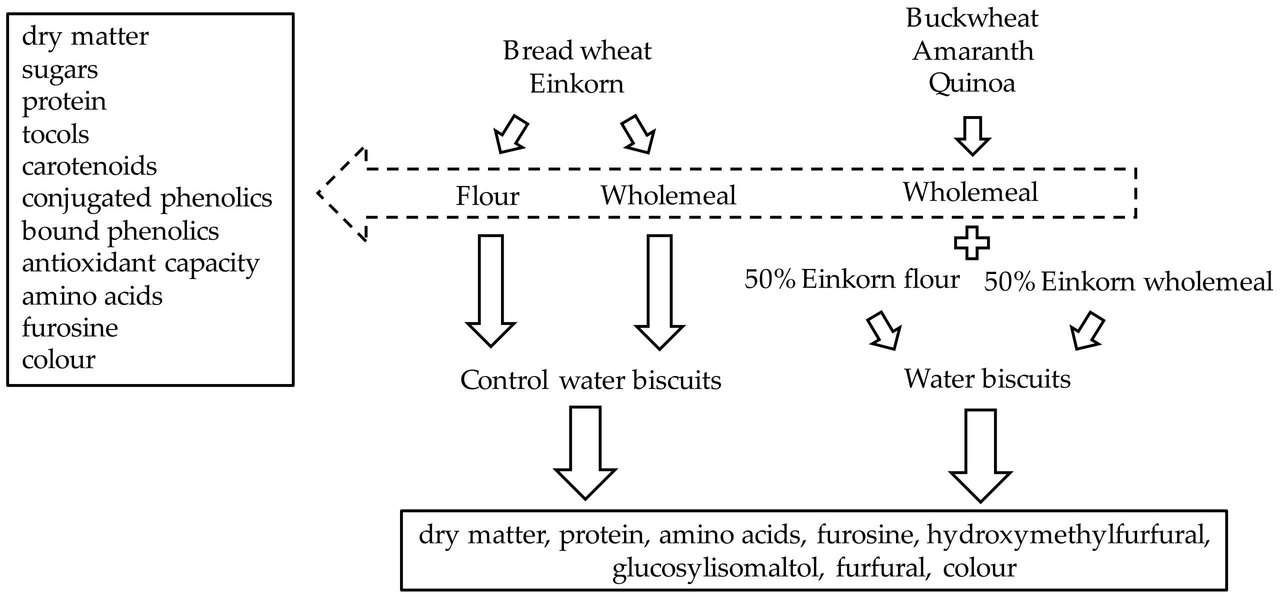

Table S1. Analysis of variance (ANOVA) antioxidants content and antioxidant capacity of the whole meals of buckwheat, amaranth, quinoa, bread wheat and einkorn (***, p≤0.001).

	**Species**	**Error**
Degrees of freedom	4	5
**Tocols**		
α-tocopherol	193.1***	0.10
β-tocopherol	289.4***	0.11
γ-tocopherol	2372.5***	0.02
δ-tocopherol	10.20***	0.01
α-tocotrienol	57.7***	0.01
β-tocotrienol	937.8***	0.68
Total	573.5***	1.48
**Carotenoids**		
(α+β)-carotene	0.06***	0.01
β-cryptoxanthin	0.01***	0.01
Lutein	7.07***	0.01
Zeaxanthin	0.30***	0.01
Total	9.12***	0.01
**Soluble conjugated phenolic acids**		
p-hydroxybenzoic acid	1104.5***	0.60
Vanillic acid	2439.3***	0.54
Syringic acid	20.0***	0.07
Syringaldehyde	10834.1***	1.81
p-coumaric acid	497.8***	0.11
Ferulic acid	1121.3***	0.74
Total	14971.7***	4.27
**Insoluble bound phenolic acids**		
p-hydroxybenzoic	165.9***	0.18
Vanillic acid	13.9***	0.03
Syringic acid	13.9***	0.11
Syringaldehyde	741.3***	0.17
p-coumaric acid	247.3***	0.89
Ferulic acid	217814.0***	272.0
Total	213217.0***	289.2
**Antioxidant capacity**		
$ABTS_{BuOH}$	98.0***	0.02
$FRAP_{MeOH:HCl}$	379.4***	0.06
$FRAP_{BuOH}$	54.0***	0.06

BuOH, saturated butanol extracts; MeOH:HCl, acidified methanol extracts

Table S2. Analysis of variance (ANOVA) of sugars, ash, protein and furosine content, and colour coordinates of the whole meals of buckwheat, amaranth, quinoa, bread wheat and einkorn and flour of bread wheat and einkorn (***, p≤0.001).

	**Species**	**Error**
Degrees of freedom	6	7
Sugars		
Fructose	0.01***	0.01
Glucose	0.01***	0.01
Maltose	0.01***	0.01
Sucrose	0.72***	0.01
Protein	6.71***	0.01
Furosine	344.4***	1.66
Degrees of freedom	6	14
Colour		
*L**	174.0***	0.84
*a**	6.09***	0.02
*b**	20.24***	0.06
Chroma	20.25***	0.07
Hue angle	136.9***	0.43

Table S3. Analysis of variance (ANOVA; mean square) of the heat damage indices and colour coordinates in water biscuits from whole meal or refined flour of einkorn or bread wheat and from 50:50 blends of einkorn whole meal or refined flour and pseudocereals whole meal (* $p \leq 0.05$, *** $p \leq 0.001$).

	Type of flour (T)	Blend (B)	T x B	Error
Degrees of freedom	1	4	4	10
Furosine	1245.0*	96751.6***	5049.3***	240.8
GLI	0.72***	0.28***	0.24***	0.002
HMF	12.31***	11.57***	10.14***	0.03
Furfural	71.82***	23.03***	24.44***	0.13
Degrees of freedom	1	4	4	20
*L**	509.7***	576.3***	47.9***	0.56
*a**	92.7***	46.95***	11.25***	0.09
*b**	8.70***	185.9***	4.36***	0.22
Chroma	1.68*	192.4***	4.33***	0.24
Hue angle	400.4***	155.6***	47.0***	0.21
